# Ratiometric Colorimetric Detection of Nitrite Realized by Stringing Nanozyme Catalysis and Diazotization Together

**DOI:** 10.3390/bios11080280

**Published:** 2021-08-18

**Authors:** Mengzhu Wang, Peng Liu, Hengjia Zhu, Bangxiang Liu, Xiangheng Niu

**Affiliations:** 1 Institute of Green Chemistry and Chemical Technology, School of Chemistry and Chemical Engineering, Jiangsu University, Zhenjiang 212013, China; wangmengzhu2018@126.com (M.W.); lp18852858622@163.com (P.L.); lbx15912338469@163.com (B.L.); 2 School of Agricultural Engineering, Jiangsu University, Zhenjiang 212013, China; 18260622650@163.com; 3 Key Laboratory of Functional Molecular Solids, Ministry of Education, Anhui Normal University, Wuhu 241002, China; 4 State Key Laboratory of Urban Water Resource and Environment, Harbin Institute of Technology, Harbin 150090, China

**Keywords:** nitrite, ratiometric colorimetric detection, nanozyme catalysis, diazotization reaction, food analysis

## Abstract

Due to the great threat posed by excessive nitrite in food and drinking water to human health, it calls for developing reliable, convenient, and low-cost methods for nitrite detection. Herein, we string nanozyme catalysis and diazotization together and develop a ratiometric colorimetric approach for sensing nitrite in food. First, hollow MnFeO (a mixture of Mn and Fe oxides with different oxidation states) derived from a Mn-Fe Prussian blue analogue is explored as an oxidase mimic with high efficiency in catalyzing the colorless 3,3′,5,5′-tetramethylbenzidine (TMB) oxidation to blue TMBox, presenting a notable signal at 652 nm. Then, nitrite is able to trigger the diazotization of the product TMBox, not only decreasing the signal at 652 nm but also producing a new signal at 445 nm. Thus, the analyte-induced reverse changes of the two signals enable us to establish a ratiometric colorimetric assay for nitrite analysis. According to the above strategy, facile determination of nitrite in the range of 3.3–133.3 μM with good specificity was realized, providing a detection limit down to 0.2 μM. Compared with conventional single-signal analysis, our dual-signal ratiometric colorimetric mode was demonstrated to offer higher sensitivity, a lower detection limit, and better anti-interference ability against external detection environments. Practical applications of the approach in examining nitrite in food matrices were also verified.

## 1. Introduction

As an industrial salt, nitrite is extensively utilized in the food industry. It is often employed as a preservative or coloring agent in food processing [1]. However, excessive nitrite in food and drinking water poses a great threat to human health [2]. It can react with amines of amino acids to form nitrosamines, which have a recognized carcinogenic effect. In addition, consumption of food containing high-level nitrite can decrease the oxygen-carrying capacity of hemoglobin [3], possibly leading to histanoxia. Due to its high toxicity, the World Health Organization has permitted a maximum amount (≈65 μM) of nitrite in drinking water [4], and a maximum daily intake (0.06 mg/kg/day) of nitrite in food has been set by the Joint Food and Agriculture Organization/World Health Organization Expert Committee on Food Additives. Thus, developing reliable, convenient, and low-cost methods for nitrite detection is greatly significant to ensure food safety and human health.

Until today, a few approaches based on different principles have been explored and applied for nitrite analysis [1], including chromatography [5,6,7], electrochemistry [8,9,10,11,12,13], fluorometry [14,15,16,17,18,19], and colorimetry [20,21,22,23,24,25,26,27,28,29,30,31,32]. Among these methodologies, colorimetric assays attract special interest because of their superiorities of convenient signal reading, result visualization, and simple operation. For instance, Daniel et al. utilized gold nanoparticles modified with aniline and naphthalene moieties as a probe and realized the colorimetric detection of nitrite based on the target facilitating the cross-coupling of the nanoparticle probe and its distance-dependent plasmonic feature [20]. Nam et al. successfully developed a nitrite colorimetric hydrogel biosensor using the Griess reagent-based diazo-coupling reaction [23]. In spite of these advances, most of the currently developed colorimetric assays rely on the change of a single signal induced by nitrite [21,22,23,24,25,26,27,28,29,30,31,32], possibly exposing the insufficiency of sensitivity for low-level analyte detection. In addition, these single-signal assays are susceptible to interference in practical application situations. Developing multi-signal modes to improve the sensitivity of nitrite colorimetric assays and their anti-interference ability against external detection environments in practical applications is highly desired.

Since Yan’s group reported the peroxidase-like characteristic of common Fe_3_O_4_ particles [33], nanomaterials with some enzyme-mimicking features, named as ‘nanozymes’ [34], have drawn increasing attention in the past few years [35,36,37]. Compared with bioenzymes, nanozymes possess the advantages of low cost, excellent stability, and easy design and regulation. With the catalytic nature to provide amplified signals, nanozymes have found promising applications in biochemical sensing [38,39,40,41], environmental monitoring [42,43,44,45], and food analysis [46,47,48]. For nitrite detection, Liu et al. used histidine-capped gold nanoclusters as an oxidase-like nanozyme to catalyze the 3,3′,5,5′-tetramethylbenzidine (TMB) oxidation and realized both the colorimetric and electrochemical detection of nitrite based on the analyzer suppressing the above reaction [49]. Adegoke et al. developed a ternary hybrid with nitrite reductase-like catalytic activity for the colorimetric sensing of nitrite [50]. These successful examples imply the great potential of nanozymes in sensing the analyte. However, they are still based on the target-induced change of a single signal. How to use nanozyme catalysis to develop multi-signal sensing methods with enhanced sensitivity and anti-interference ability against external detection environments for nitrite detection is still a challenge.

**Scheme 1:**
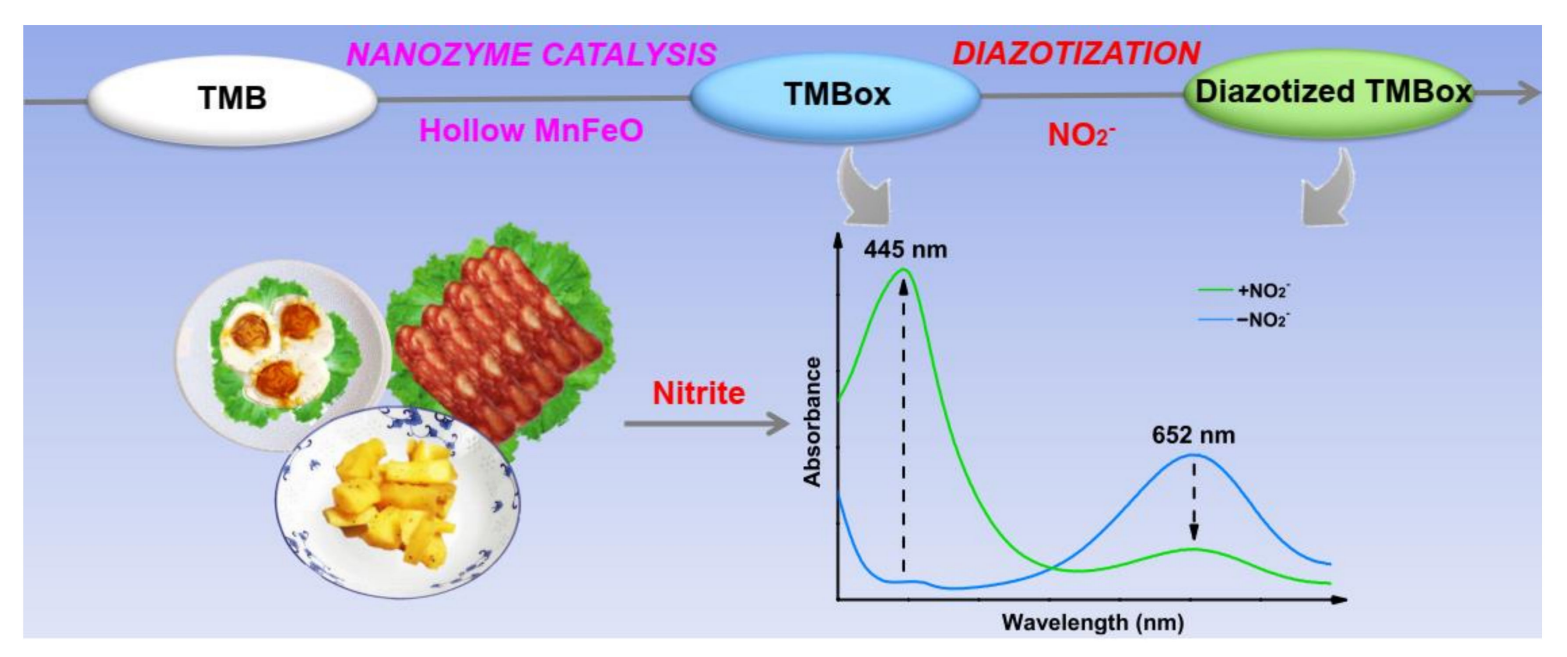
Schematic illustration of the ratiometric colorimetric detection of nitrite in food based on the cascade reaction of nanozyme catalysis and diazotization.

In the present work, we propose a ratiometric colorimetric method with high performance for nitrite detection in food by stringing nanozyme catalysis and diazotization together. As illustrated in Scheme 1, hollow MnFeO particles (a mixture of Mn and Fe oxides with different oxidation states) derived from a Mn-Fe Prussian blue analogue (PBA) are first employed as an oxidase mimic to efficiently catalyze the oxidation of colorless TMB to blue TMBox, providing a prominent UV-visible (UV-vis) absorption signal at 652 nm; then, the analyte nitrite can trigger the diazotization of the blue product TMBox, leading to the formation of diazotized TMBox with a yellow color. Interestingly, the diazotization process not only weakens the original absorption signal at 652 nm but also offers a new one at approximately 445 nm. Thus, the reverse changes of the two signals induced by nitrite enable us to develop a ratiometric colorimetric assay for nitrite sensing. On the basis of the above principle, the proposed nitrite assay provided better performance (higher sensitivity, lower detection limit, and better anti-interference ability against external detection environments) compared with traditional single-signal analysis. In addition, applications of our dual-signal assay in analyzing the target in food samples were also demonstrated.

## 2. Experimental Section

### 2.1. Chemicals and Reagents

Fe_2_(SO_4_)_3_ was obtained from Shanghai Eon Chemical Technology Co., Ltd. MnSO_4_·H_2_O, polyvinylpyrrolidone K30 (PVP), ethanol (EtOH), NaNO_2_, acetic acid (HAc), and sodium acetate (NaAc) were supplied by Sinopharm Chemical Reagent Co., Ltd. K_3_[Fe(CN)_6_], ascorbic acid (AA), and TMB were purchased from Shanghai Aladdin Reagents Co., Ltd. Thioglycolic acid (TGA) was obtained from Sigma-Aldrich. All the reagents and chemicals utilized in the study were at the analytical level. In the whole research, deionized water (DW) was utilized.

### 2.2. Preparation and Characterization of Hollow MnFeO

Hollow MnFeO was prepared according to the previous literature [51] with some modifications. In detail, (1) 0.048 mmol Fe_2_(SO_4_)_3_ and 0.373 mmol MnSO_4_·H_2_O were dissolved in a solution of 0.6 g PVP, 20 mL EtOH, and 20 mL DW. After stirring for 15 min, K_3_[Fe(CN)_6_] (20 mM, 20 mL) was slowly added for reaction at room temperature for 1 h. After that, the formed precipitate Mn-Fe PBA was washed with EtOH and DW several times; (2) 0.1 g Mn-Fe PBA was dispersed in 25 mL DW and sonicated for 20 min. Afterwards, 15 mL TGA (6 mM) was added to the above mixture for etching at room temperature for 6 h. After that, hollow Mn-Fe PBA was obtained by centrifugal separation and freeze drying; (3) hollow Mn-Fe PBA was calcinated in a tubular furnace at 400 °C in air for 1 h; thus, hollow MnFeO was formed. For comparison, Mn-Fe PBA was directly calcinated under the same conditions to obtain MnFeO.

A 6100Lab diffractometer (Shimadzu) provided X-ray diffraction (XRD) measurements. A 7001 scanning electron microscope (SEM, JEOL) and an H-7800 transmission electron microscope (TEM, Hitachi) were used to observe the morphology of materials. The pore structures were obtained by N_2_ adsorption/desorption isotherms on a Micromeritics TriStar II 3020 analyzer (Micromeritics Instrument Corporation, USA). Fourier transformed infrared (FTIR) spectra were measured by a Nicolet Nexus 470 spectrometer (USA Nicolet). X-ray photoelectron spectroscopy (XPS) measurements were accomplished by a Thermo Scientific K-Alpha instrument.

### 2.3. Oxidase-Mimicking Characteristic of Hollow MnFeO Catalyzing TMB Oxidation

The oxidase-mimicking catalytic characteristic of hollow MnFeO was studied by employing TMB as a common substrate. Concretely, 30 μL of 1 mg/mL hollow MnFeO aqueous solution and 50 μL of 5 mM TMB dissolved in ethanol were added to 1420 μL NaAc-HAc buffer (0.2 M, pH 4). The mixture was measured by an Evolution 201 UV-vis spectrophotometer (Thermo Scientific) after reaction at room temperature for 15 min. Under optimal conditions, steady-state kinetics of oxidase-mimicking hollow MnFeO catalyzing the oxidation of TMB were derived from the Michaelis–Menten fitting by recording the absorbance at 652 nm within 60 s at a 10 s interval. For each measurement, only the concentration of TMB was changed. The values of *V*_max_ and *K*_m_ could be obtained according to the following Michaelis–Menten equation:

*V* = *V*_max_[*S*]/(*K*_m_ + [*S*])

where *V* is the initial velocity, *V*_max_ is the maximum reaction velocity, [*S*] is the concentration of TMB, and *K*_m_ is the Michaelis–Menten constant.

### 2.4. Nitrite-Induced Diazotization of TMBox

To estimate the effect of nitrite on the product TMBox originating from the oxidation of TMB catalyzed by oxidase-like hollow MnFeO, 30 μL hollow MnFeO aqueous solution (1 mg/mL) and 50 μL TMB solution (5 mM, dissolved in ethanol) were added to 1405 μL NaAc-HAc buffer (0.2 M, pH 4). After reaction at room temperature for 15 min, 15 μL nitrite aqueous solution (10 mM) was added to the above mixture. After further reaction at room temperature for 40 min, the solution was tested by UV-vis.

### 2.5. Ratiometric Colorimetric Analysis of Nitrite

The feasibility of our ratiometric colorimetric method for nitrite detection was verified by recording the response of the analyte at different concentrations. In detail, 25 μL hollow MnFeO aqueous solution (1 mg/mL) and 30 μL TMB solution (5 mM, dissolved in ethanol) were added to NaAc-HAc buffer (0.2 M, pH 4). After reaction at room temperature for 15 min, different concentrations of nitrite aqueous solutions (including 3.3 μM, 6.7 μM, 10 μM, 13.3 μM, 20 μM, 33.3 μM, 50 μM, 66.7 μM, 83.3 μM, 100 μM, 120 μM, 133.3 μM, 166.7 μM, 186.7 μM, 200 μM, and 233.3 μM) were added to the above mixture. After incubation at room temperature for 40 min, the UV-vis absorption signal was recorded.

The anti-interference test was performed to assess the specificity of our method. In this work, possible effects of common ions, including K^+^, Mg^2+^, Ba^2+^, Fe^3+^, Ni^2+^, Cl^−^, CO_3_^2−^, SO_4_^2−^, PO_4_^3−^, and NO_3_^−^, and biomolecules, including glucose and glutamic acid (Glu), on the hollow MnFeO+TMB system were tested. In detail, 25 μL hollow MnFeO aqueous solution (1 mg/mL) and 30 μL TMB solution (5 mM, dissolved in ethanol) were added to NaAc-HAc buffer (0.2 M, pH 4). After reaction at room temperature for 15 min, different species were added to the above mixture. After incubation at room temperature for 40 min, the UV-vis absorption signal was recorded.

To assess the practicability of our approach, it was employed to analyze nitrite in real food matrices, including sausage, pickle, and salted eggs. These samples were obtained from a local market (Zhenjiang, PR China). For sample treatment, 5 g of homogenized sample was placed in a 250 mL conical flask, and 12.5 mL of saturated borax solution (50 g/L) and 150 mL DW (70 °C) were added and stirred well. The sample was heated in a boiling water bath for 15 min before being transferred to a cold water bath and cooled to room temperature. Next, the above solution was transferred to a 200 mL flask, and 5 mL of K_4_[Fe(CN)_6_] solution (106 g/L) was added and shaken well. After that, 5 mL of 220 g/L zinc acetate aqueous solution was added to precipitate proteins in food. Finally, DW was added to the scale line and placed for 30 min. After the upper fat layer was eliminated, the mixture was filtered by a common filter paper to gain the sample solution. Then, the obtained sample solution was tested according to the above protocol. Meanwhile, a commercial food nitrite content detection kit (Beijing Solarbio Science & Technology Co., Ltd., Product No. BC1490) was used as a standard reference tool (GB 5009.33-2016) to verify the reliability of our assay.

## 3. Results and Discussion

### 3.1. Synthesis and Characterization of Hollow MnFeO

**Figure 1:**
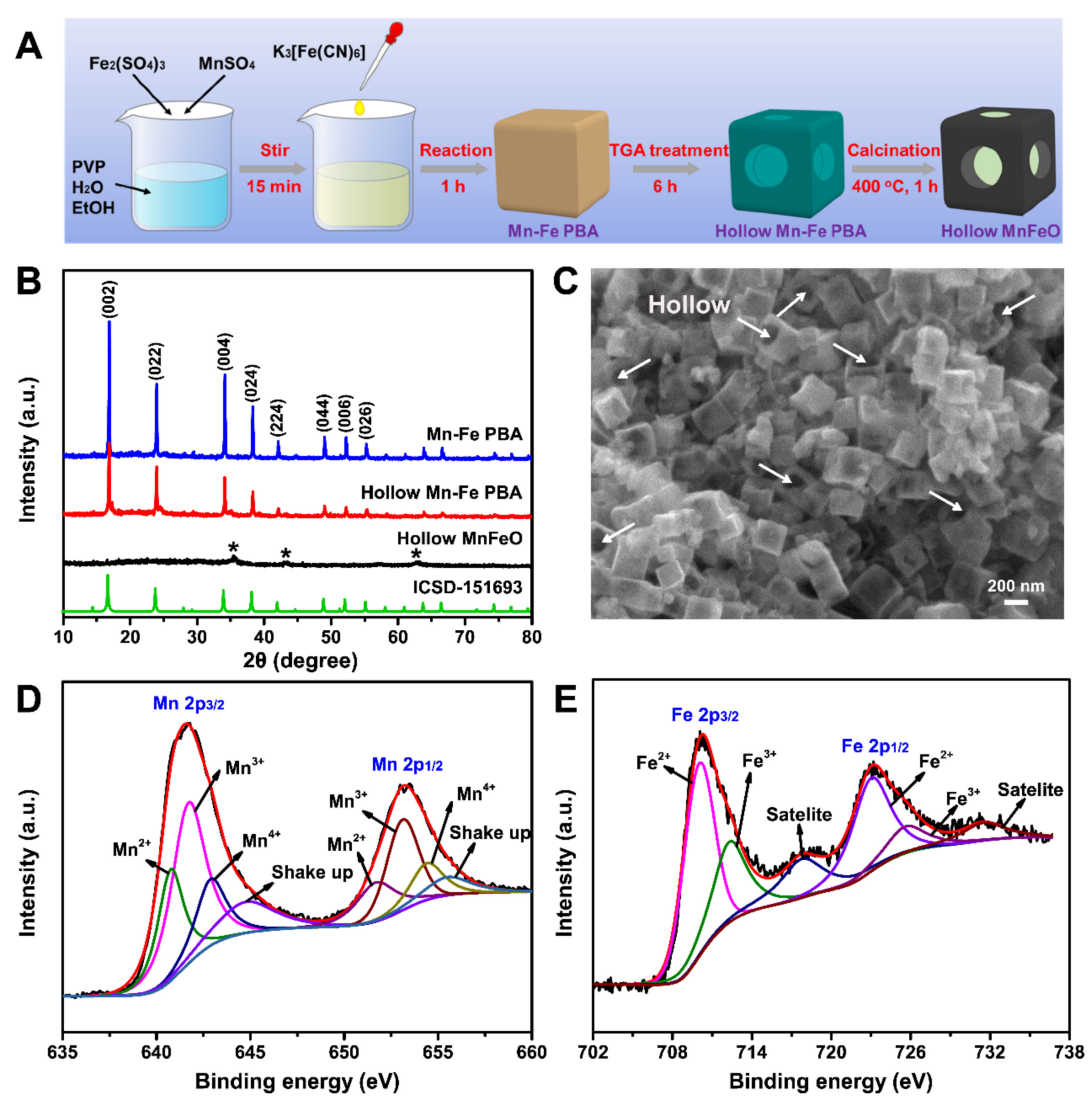
(**A**) illustrates the preparation process of hollow MnFeO. (**B**) compares XRD profiles of different materials. (**C**) depicts the SEM image of hollow MnFeO. (**D**,**E**) present fine Mn 2p and Fe 2p XPS of hollow MnFeO, respectively.

In the present work, hollow MnFeO was obtained via a three-step process according to the literature [51]. As illustrated in Figure 1A, Fe^3+^, Mn^2+^ and [Fe(CN)_6_]^3−^ are used as precursors to form a Mn-Fe PBA via co-precipitation, where PVP is employed as a surfactant to stabilize the solid particles formed. After that, TGA is used to etch these particles to get hollow Mn-Fe PBA. Finally, hollow Mn-Fe PBA is calcinated in air at 400 °C; thus, the desired material hollow MnFeO is gained.

To verify the successful preparation of hollow MnFeO, a series of characterization experiments were performed. As compared in Figure 1B, the obtained Mn-Fe PBA with a cubic shape (Figure S1A, Supplementary Materials) presents the characteristic peaks of Mn_4_[Fe(CN)_6_]_2.667_ (ICSD-151693) [51]. The FTIR spectrum (Figure S5, Supplementary Materials) of Mn-Fe PBA contains two bands of *ν*(CN), which are attributed to Mn^III^–NC–Fe^II^ (around 2070 cm^−1^) and Mn^II^–NC–Fe^III^ (around 2149 cm^−1^), indicating the existence of mixed phases in it [52]. After TGA treatment, the core of these cubic particles is first dissolved by TGA, while the shell remains intact [53], thus forming hollow Mn-Fe PBA cages (Figure S1B, Supplementary Materials). In XRD (Figure 1B), the diffraction peaks at 16.9°, 23.9°, 34.1°, 38.3°, 42.1°, 49.0°, 52.2° and 55.3° are ascribed to the (002), (022), (004), (024), (224), (044), (006), and (026) crystal planes of Mn-Fe PBA (ICSD-151693), respectively [51]. The result shows that the Mn-Fe PBA composite is successfully prepared. The characteristic diffraction signals are weakened slightly observed in hollow Mn-Fe PBA. As indicated by the *ν*(CN) signal of Mn^III^–NC–Fe^II^ in FTIR (Figure S5, Supplementary Materials), the residual shell mainly consists of Mn^III^_2_[Fe^II^(CN)_4_]_3_. Given that the core is etched by TGA, the *ν*(CN) peak of Mn^II^–NC–Fe^III^ disappears. After calcination in air at a high temperature (400 °C), these characteristic diffraction peaks disappear in hollow MnFeO (Figure 1B). Instead, weak signals attributed to Fe and Mn oxides (marked with asterisks) appear in comparison with hollow Mn-Fe PBA. Both the SEM image (Figure 1C) and TEM image (Figure S3, Supplementary Materials) demonstrate that the calcination process does not change the morphology significantly, and a cage structure still remains in these hollow MnFeO particles. The EDS result (Figure S2A, Supplementary Materials) reveals the presence of Mn, Fe, and O in the hollow MnFeO material, and the elemental mapping analysis (Figure S2B–E, Supplementary Materials) clearly indicates that the Mn, Fe, and O elements are uniformly distributed in the material. In the FTIR proof, a broad signal at approximately 600 cm^−1^ is observed (Figure S5, Supplementary Materials), which should be assigned to the vibrations of Mn–O and Fe–O [54]. According to N_2_ adsorption/desorption isotherms (Figure S4A, Supplementary Materials), the adsorption and desorption curves have an obvious hysteresis ring, indicating that hollow MnFeO has a mesoporous structure. The specific surface area (Figure S4A, Supplementary Materials), pore volume, and pore diameter (Figure S4B, Supplementary Materials) of hollow MnFeO are 176.040 m²/g, 0.232 cm^3^/g, and 4.497 nm, respectively. Furthermore, XPS was employed to investigate the chemical composition of hollow MnFeO. The full XPS indicates the presence of Mn, Fe, and O in hollow MnFeO again (Figure S6, Supplementary Materials). According to the XPS result, the atomic ratio of Fe to Mn in the obtained hollow MnFeO is approximately 1:2. In the fine Mn 2p XPS (Figure 1D), the peaks of Mn 2p_1/2_ and Mn 2p_3/2_ are centered at 653.2 eV and 641.6 eV, respectively, which can further be deconvoluted to Mn^II^, Mn^III^, and Mn^IV^ with corresponding shake-up signals. As shown in Figure 1E, the XPS spectrum of Fe 2p shows two main peaks with binding energies at 723.9 eV and 710.9 eV, corresponding to Fe 2p_1/2_ and Fe 2p_3/2_, respectively. The deconvoluted Fe 2p XPS indicates the coexistence of Fe^II^ and Fe^III^. All the results are in agreement with the literature [52], demonstrating the successful synthesis of the desired material hollow MnFeO.

### 3.2. Oxidase-Mimicking Characteristic of Hollow MnFeO Catalyzing the Oxidation of TMB to TMBox

**Figure 2:**
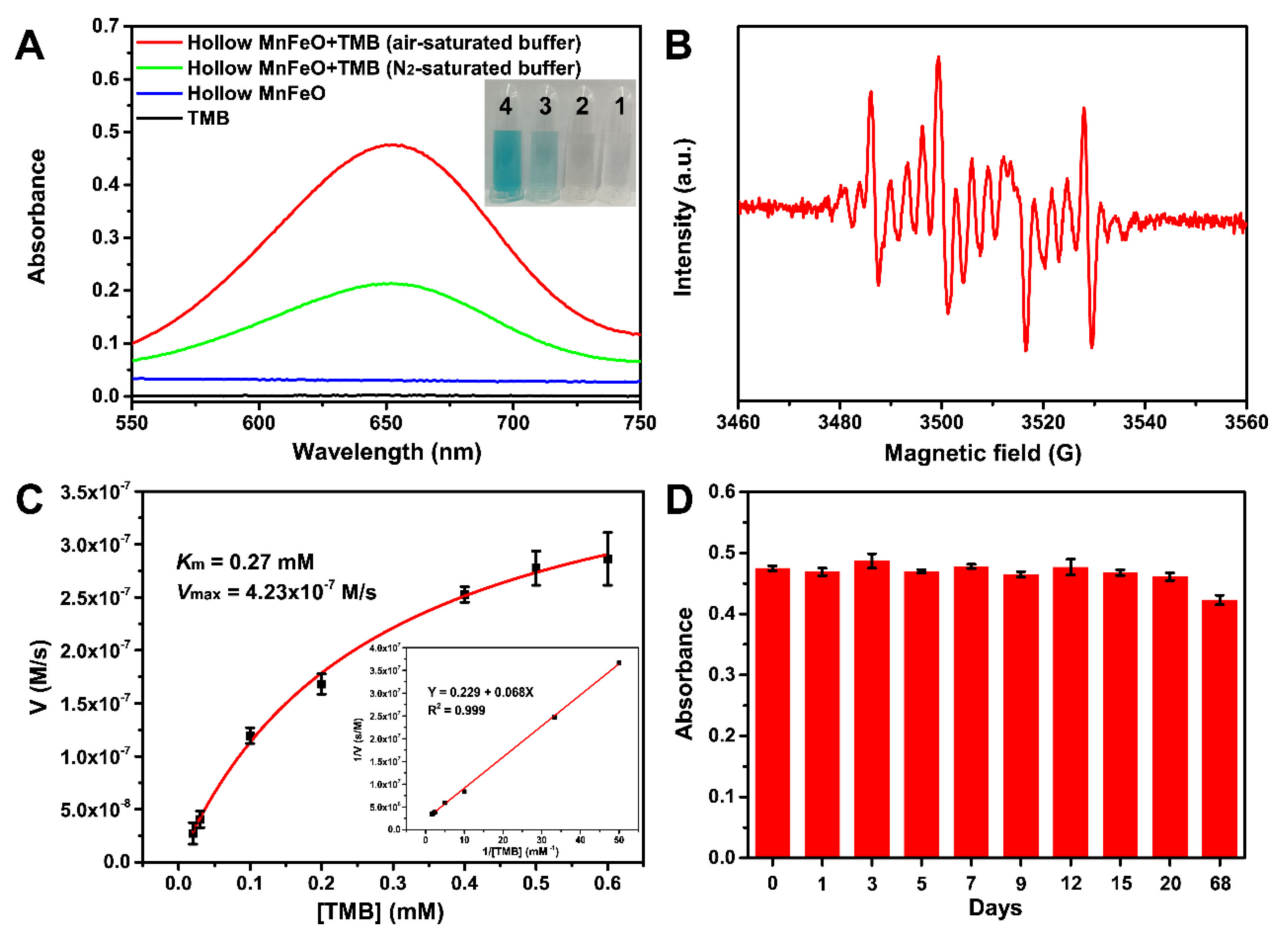
(**A**) compares UV-vis spectra of different systems, and the inset is the corresponding photograph (1—TMB; 2—Hollow MnFeO; 3—Hollow MnFeO+TMB in N_2_-saturated buffer; 4—Hollow MnFeO+TMB in air-saturated buffer). (**B**) shows the EPR spectrum of the hollow MnFeO solution with 5,5-dimethyl-1-pyrroline-1-oxide as a capturer. (**C**) records kinetic plots of hollow MnFeO catalyzing the TMB chromogenic reaction, and the inset shows the corresponding double reciprocal graph. (**D**) demonstrates the excellent stability of hollow MnFeO as an oxidase mimic stored in DW at room temperature.

Given the presence of mixed valences of Mn^2+^, Mn^3+^, and Mn^4+^ as well as Fe^2+^ and Fe^3+^ (Figure 1D,E), the obtained hollow MnFeO is supposed to have some enzyme-like catalytic activities. To demonstrate this hypothesis, TMB was employed as a common substrate to study the oxidase-like feature of the material. As compared in Figure 2A, hollow MnFeO has no obvious background color at such a concentration (20 μg/mL), and the substrate cannot be directly oxidized by dissolved O_2_. However, when a small amount (20 μg/mL) of hollow MnFeO is added to the TMB solution with dissolved O_2_, a remarkable color reaction appears rapidly, providing a prominent UV-vis absorption peak at around 652 nm. The colorless solution becomes blue in a short period of time. Such a color change is attributed to the oxidation of the colorless substrate TMB to its oxide TMBox, implying the ability of hollow MnFeO to trigger the redox reaction of dissolved O_2_ and TMB. To confirm that the hollow MnFeO added indeed acts as a catalyst but not as an oxidizing agent to induce the oxidation of TMB, the reaction occurring in air- or N_2_-saturated buffers was compared [55,56]. In comparison with the case in air-saturated solution, in N_2_-saturated buffer the TMB oxidation reaction is significantly suppressed (Figure 2A). Such a comparison implies that the reaction relies on the presence of O_2_. The result confirms the role of hollow MnFeO as an oxidase-like nanozyme catalyzing the redox reaction between dissolved O_2_ and TMB [55]. The electron paramagnetic resonance (EPR) result (Figure 2B) implies the production of superoxide radicals during the reaction. Thus, it is reasonably deduced that dissolved O_2_ first receives an electron under the catalysis of oxidase-mimicking hollow MnFeO to produce superoxide anion radicals, and the latter with a remarkable oxidizing capacity further induces the oxidation of the colorless substrate TMB to blue TMBox [57,58].

To further understand the oxidase-like feature of hollow MnFeO, factors affecting the oxidation of TMB catalyzed by the nanozyme were studied. As shown in Figure S7 (Supplementary Materials), buffer pH has a significant impact on the catalytic color reaction, where in medium acidic environments, the nanozyme presents remarkable catalytic activity, while under neutral conditions, the activity is decreased sharply, in agreement with previously reported oxidase mimics [46,55,59]. Given that at pH 4, the nanozyme shows a maximum catalytic ability, the following experiments are performed in the buffer pH 4. As expected, with the increase of the nanozyme amount added, the absorbance (652 nm) of the hollow MnFeO+TMB system also increases (Figure S8, Supplementary Materials). The time-dependent absorbance change (Figure S9, Supplementary Materials) indicates that after reaction for 10 min, the absorbance (652 nm) becomes saturated. To ensure that all the substrate added is catalytically oxidized completely, in the following study, a reaction time of 15 min is utilized.

Under optimal conditions, the catalytic kinetic behavior of oxidase-mimicking hollow MnFeO was investigated at room temperature [60]. As depicted in Figure 2C, the initial reaction rate increases with TMB concentration according to the Michaelis–Menten kinetic mode. As compared in Table S1 (Supplementary Materials), the apparent Michaelis–Menten constant (*K*_m_) value of 0.27 mM is lower than that of CeO_2_ [61], Pt nanoclusters [62], and Mn_3_O_4_ with oxygen vacancies [57], which are indicative of a stronger affinity of TMB toward the surface of hollow MnFeO. The maximum velocity (*V*_max_) value of hollow MnFeO is found to be comparable or even larger than that of previously developed materials [46,57,61], demonstrating the high catalytic activity of hollow MnFeO as an oxidase mimic.

To better highlight the oxidase-like activity of hollow MnFeO, a comparison of the nanozyme with its counterparts catalyzing the TMB oxidation reaction under the same conditions was further made. As indicated in Figure S10 (Supplementary Materials), neither Mn-Fe PBA nor hollow Mn-Fe PBA exhibits any obvious oxidase-like activity toward the reaction. This may be due to the active Mn and Fe centers being coordinated by the –CN– ligand. Compared with the MnFeO counterpart, hollow MnFeO can exhibit a higher activity to catalyze the oxidation of TMB (Figure S11, Supplementary Materials). This is because the latter is able to expose more active surfaces for substrate access and reaction.

In comparison with natural bioenzymes, an attractive merit of inorganic materials as enzyme mimics is their excellent stability. To check the catalytic stability of hollow MnFeO, it was stocked in DW at room temperature for a period of time, and the oxidase-like activity was then tested under standard conditions. As demonstrated by Figure 2D, different from natural enzymes losing their activity gradually, hollow MnFeO shows no obvious change of the activity after a long-term storage. This result indicates the good oxidase-mimicking catalytic stability of the nanozyme.

### 3.3. Nitrite Induces the Diazotization of TMBox

**Figure 3:**
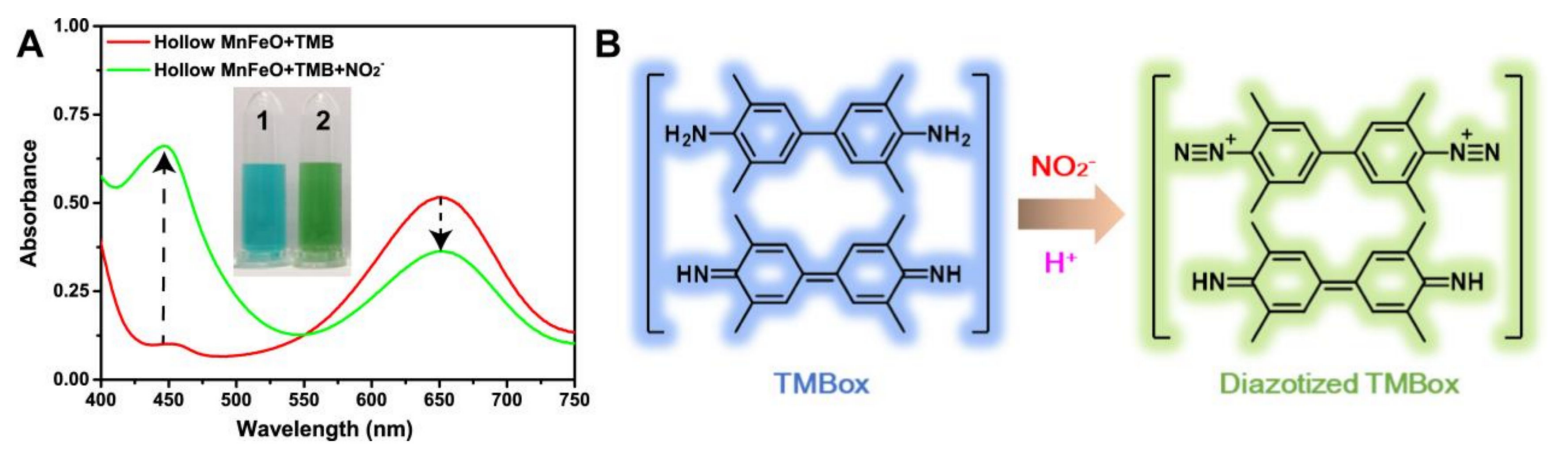
(**A**) records the effect of nitrite on the hollow MnFeO+TMB system, and the inset is the corresponding photograph (1—Hollow MnFeO+TMB; 2—Hollow MnFeO+TMB+NO_2_^−^). (**B**) illustrates the mechanism for the phenomenon observed in (**A**).

Interestingly, it is observed that the addition of nitrite can change the color of the hollow MnFeO+TMB solution. As presented in Figure 3A, the hollow MnFeO+TMB system exhibits a blue color because of the oxidation of TMB to TMBox under the catalysis of hollow MnFeO. When a small amount (100 μM) of nitrite is added, the solution becomes green gradually. Correspondingly, the UV-vis absorption signal at 652 nm attributing to TMBox decreases when nitrite exists, while a new remarkable signal appears at approximately 445 nm. Given that there is no obvious interaction between nitrite and the nanozyme (Figure S12, Supplementary Materials), the above phenomenon can only be assigned to the reaction between TMBox and nitrite. As illustrated in Figure 3B, since there is an aromatic primary amine group in TMBox, it can react with NO_2_^−^ in the acidic environment (pH 4) to perform a typical diazotization reaction. As a result, the consumption of blue TMBox decreases the original signal at 652 nm, and the generated diazotized TMBox provides the new absorption peak at 445 nm. Furthermore, some control experiments were designed to demonstrate that the diazotization reaction indeed occurs. As demonstrated in Figure S13 (Supplementary Materials), a high concentration of the substrate TMB can be oxidized to blue TMBox under the catalysis of hollow MnFeO, and a low concentration of TMB can further be catalytically overoxidized to a yellow product (overoxidized TMBox). When a small amount of AA is added, both blue TMBox and yellow overoxidized TMBox can be re-reduced to colorless TMB. However, when NO_2_^−^ is added, it interacts with TMBox via the diazotization reaction, forming diazotized TMBox with a green color. When AA is further added, the formed diazotized TMBox cannot be re-reduced to colorless TMB. This result also demonstrates that the typical diazotization reaction indeed occurs between nitrite and the TMBox species formed.

What should be stated is that the species nitrite can directly react with the original substrate TMB. As demonstrated in Figure S14 (Supplementary Materials), when nitrite and TMB are incubated together at room temperature with no addition of the nanozyme, the UV-vis spectrum of the mixture provides a notable peak at 652 nm, which is assigned to the TMB oxidation to TMBox. Such a phenomenon involves the redox reaction between nitrite and TMB [63]. Additionally, an absorption signal centered at 439 nm is observed, which is due to the diazotization reaction of nitrite and TMB [30].

Importantly, the cascade of oxidase-like catalysis and diazotization has good specificity toward the species nitrite. Figure S15 (Supplementary Materials) compares the responses of common ions including K^+^, Mg^2+^, Ba^2+^, Fe^3+^, Ni^2+^, Cl^−^, CO_3_^2−^, SO_4_^2−^, PO_4_^3−^, and NO_3_^−^ and biomolecules including glucose and glutamic acid (Glu) toward the hollow MnFeO+TMB system. It is found that only nitrite can trigger the generation of the signal at 445 nm, which is indicative of the system’s good selectivity toward the target. Such specificity offers us the basis for nitrite detection.

### 3.4. High-Performance Ratiometric Colorimetric Analysis of Nitrite

Given that nitrite is able to induce the inverse changes of the two signals (652 nm and 445 nm) in the hollow MnFeO+TMB system with good specificity (Figure 3A and Figure S15, Supplementary Materials), it provides us a dual-signal ratiometric colorimetric method to quantitatively analyze nitrite. To better detect the analyte, the reaction time of TMBox and nitrite was optimized. As depicted in Figure S16 (Supplementary Materials), the ratio (A_652_/A_445_) of the two signals at 652 nm and 445 nm decreases with the increase of reaction time, until a saturation effect gradually appears after 40 min. As a result, a reaction time of 40 min is employed for nitrite detection.

**Figure 4:**
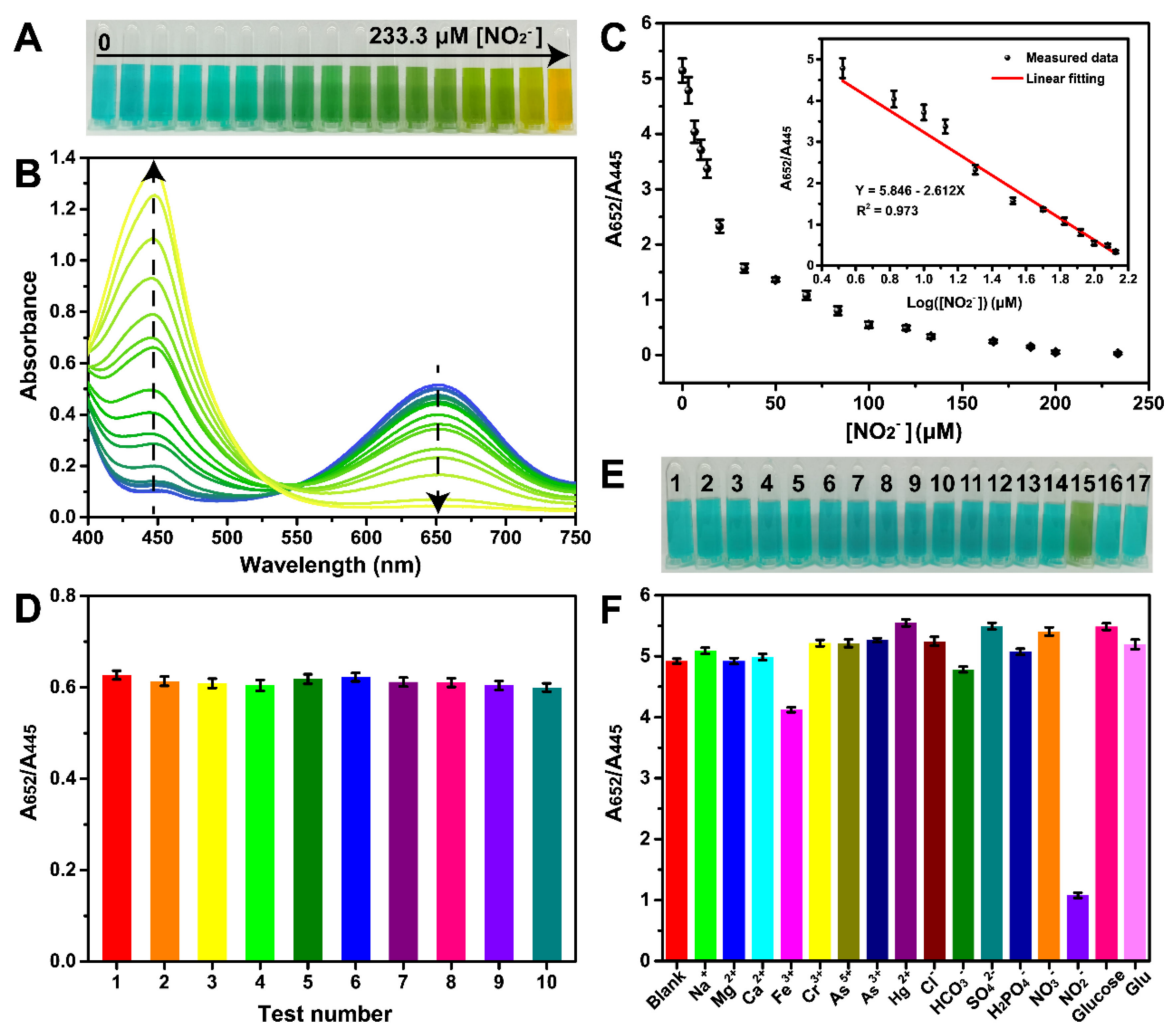
(**A**) is the photograph of the hollow MnFeO+TMB system with the presence of increasing nitrite (the concentration of nitrite increases from 0 to 233.3 μM). (**B**) displays UV-vis spectra of the hollow MnFeO+TMB+NO_2_^−^ system with nitrite at different levels. (**C**) presents the relationship of the response of nitrite and its concentration (the inset shows the corresponding linear relationship). (**D**) displays the response of the hollow MnFeO+TMB+NO_2_^−^ system in repetitive tests (the concentration of NO_2_^−^ is 100 μM). (**E**) is the photograph of the responses of various species toward the hollow MnFeO+TMB system (1—Blank, 2—Na^+^, 3—Mg^2+^, 4—Ca^2+^, 5—Fe^3+^, 6—Cr^3+^, 7—As^5+^, 8—As^3+^, 9—Hg^2+^, 10—Cl^−^, 11—HCO_3_^2−^, 12—SO_4_^2−^, 13—H_2_PO_4_^−^, 14—NO_3_^−^, 15—NO_2_^−^, 16—Glucose and 17—Glu), and (**F**) displays UV-vis spectra of the responses of various species toward the hollow MnFeO+TMB system (the concentration of NO_2_^−^ is 50 μM, and the concentrations of other species are 100 μM).

Under optimal conditions, nitrite was quantitatively determined by our ratiometric colorimetric method. As presented in Figure 4A, with the increase of the amount of nitrite added, the color of the hollow MnFeO+TMB solution undergoes a blue–green–yellow change. Correspondingly, the consumption of blue TMBox results in the decrease of the signal at 652 nm when nitrite is increased (Figure 4B). In contrast, the generation of diazotized TMBox leads to the increase of the signal at 445 nm when more nitrite is added. By taking the A_652_/A_445_ ratio as an indicator, it gradually decreases along with the increase of nitrite concentration (Figure 4C). A good linear relationship between A_652_/A_445_ and the logarithm of nitrite level is further found in the concentration scope of 3.3–133.3 μM, and the equation can be written as A_652_/A_445_ = 5.846–2.612 Log([NO_2_^−^]) (R^2^ = 0.973). The limit of detection (LOD) is down to 0.2 μM based on the rule of three-fold signal-to-noise (S/N = 3). Given that TMB can directly react with nitrite (Figure S14, Supplementary Materials), it was also used to directly detect the analyte and as a counterpart for comparison. As demonstrated in Figure S14 (Supplementary Materials), the interaction of TMB and nitrite involves two independent reactions, namely the redox reaction between TMB and nitrite and their diazotization. Given that the generation of both the signals at 652 nm and 439 nm needs the consumption of nitrite, it is impossible to fabricate a dual-signal ratiometric detection mode. As demonstrated by the experimental result (Figure S17A, Supplementary Materials), although the absorption signal at 439 nm increases along with the concentration of nitrite, the change of the signal at 652 nm is undisciplined. By taking the signal at 439 nm as an indicator, a linear relationship is found in the concentration scope of 10–233.3 μM (Figure S17B, Supplementary Materials), and the LOD is calculated to be 0.9 μM. As demonstrated, the proposed dual-signal ratiometric colorimetric detection mode enabled by the cascade of oxidase-like catalysis and diazotization can perform better than the single-signal one in terms of sensitivity and detection limit. In comparison with single-signal methods developed by using TMB as a reagent (Table S2, Supplementary Materials), our ratiometric strategy is also superior in analytical performance. More importantly, the detection limit and sensitivity of our ratiometric approach are quite enough for the monitoring of nitrite in food matrices and drinking water (the maximum amount of nitrite in drinking water is limited to be ≈65 μM). In addition, our ratiometric colorimetric sensor can provide comparable or even better detection limit and range compared with previously reported methods (Table S3, Supplementary Materials), which is indicative of its great potential in practical applications again.

Our ratiometric colorimetric strategy can provide excellent reproducibility. As demonstrated in Figure 4D, in ten parallel tests, the method provides no obvious difference in the response of nitrite, with a relative standard deviation (RSD) of only 1.4%. This result well indicates the good repeatability of our assay. Such repeatability should be due to the inherent nature of the ratiometric mode, where the independent two signals provide self-calibration for high-reliability detection [64].

As demonstrated in Figure S15 (Supplementary Materials), only nitrite can provide a specific response toward the hollow MnFeO+TMB system. To further verify the specificity of our assay toward the analyte, the A_652_/A_445_ ratio of the system with the presence of various potential interferents was calculated and compared. As shown in Figure 4E, only the target nitrite leads to a remarkable change of the solution from blue to yellow-green. Correspondingly, a remarkable decrease of the A_652_/A_445_ value is observed in nitrite (Figure 4F), while other common species, including common ions and biomolecules, have no significant effect. The result confirms the good selectivity of our method for nitrite detection again.

Finally, to assess the practicability of our method, it was applied to analyte nitrite in complex food matrices, including sausage, pickle, and salted eggs. The pretreatment process of these samples has been described in the Experimental section. In addition, a commercial assay kit based on the Griess reagent-based diazo-coupling reaction was used as a reference tool (GB 5009.33-2016) to detect these samples. The linear detection equation obtained by the commercial kit can be written as A_540_ = 0.01221 + 0.00064[NO_2_^−^] (R^2^ = 0.974) in the concentration scope of 3–33 μM (Figure S18, Supplementary Materials). Obviously, the sensitivity of the commercial kit is much lower than that of our proposed method. As compared in Table 1, our ratiometric method with high sensitivity can detect low-level nitrite in original samples, while the commercial kit cannot analyte the target in these samples. Except for the salted egg samples, similar results to those of the reference kit indicate the good reliability of our method for nitrite analysis in spiked samples. The recovery tests suggest better reliability and anti-interference ability against external detection environments of our proposed method than the reference one. These results well demonstrate the good practicability of our method. With this, it is supposed to have promising applications in food safety management and other fields.

## 4. Conclusions

As summarized, in this work, we have developed a ratiometric colorimetric method via stringing oxidase-mimicking catalysis and diazotization together for the high-performance detection of nitrite. Compared with conventional single-signal methods for nitrite analysis, the target-induced dual-signal changes have offered higher sensitivity, lower detection limit, and better anti-interference ability against external detection environments. Reliable use of our approach in analyzing nitrite in food matrices has also been demonstrated. With high performance, low cost, and simple operation, the proposed assay is believed to have a bright future in food safety and other fields. What should be stated is that in spite of the high sensitivity and wide detection range, the currently proposed method needs a relatively long time for detection. This is mainly because the diazotization of TMBox in such an acidic buffer (pH 4) needs a relatively long time (40 min) for reaction. How to shorten the detection time while retaining good performance still needs further study.

## Figures and Tables

**Table 1:** Results of our ratiometric colorimetric method in detecting nitrite in food samples.

Sample	Spiked (μM)	Detected by Our Method (μM)	RSD (%, N = 3)	Recovery Rate (%)	Detected by the Commercial Kit	RSD (%, N = 3)	Recovery Rate (%)
Sausage	0	0.74	9.9	NA ^a^	ND ^b^	NA	NA
	20	20.57	1.2	99.2	21.33	0.2	106.6
Pickle	0	0.99	3.7	NA	ND	NA	NA
	20	20.82	1.6	99.2	21.72	3.8	108.6
Salted egg yolk	0	0.77	3.6	NA	ND	NA	NA
	20	20.65	0.7	99.4	18.77	1.0	93.8
Salted egg white	0	1.08	3.1	NA	ND	NA	NA
	20	21.00	1.6	99.6	16.21	3.9	81.0

^a^ = not applicable; ^b^ = not detected.

## Supplementary Materials

The following are available online at https://www.mdpi.com/article/10.3390/bios11080280/s1, Figure S1: SEM images of Mn-Fe PBA (A) and hollow Mn-Fe PBA (B). Figure S2: EDS (A) and elemental mapping images (B–E) of hollow MnFeO. Figure S3: TEM image of hollow MnFeO. Figure S4: N_2_ adsorption/desorption curves (A) of hollow MnFeO and its pore size distribution (B). Figure S5: Comparison of FTIR spectra of different materials. Figure S6: Full XPS of hollow MnFeO. Figure S7: Effect of buffer pH on hollow MnFeO catalyzing the TMB chromogenic reaction. Figure S8: The absorbance (652 nm) of the hollow MnFeO + TMB system increases along with the amount of hollow MnFeO used. Figure S9: Absorbance (652 nm) change of the hollow MnFeO+TMB system over reaction time. Figure S10: Neither Mn-Fe PBA nor hollow Mn-Fe PBA shows oxidase-like activity to catalyze the TMB chromogenic reaction. Figure S11: Oxidase-like activity comparison of MnFeO and hollow MnFeO catalyzing the TMB chromogenic reaction. Figure S12: No chromogenic reaction occurs between hollow MnFeO and nitrite. Figure S13: Ascorbic acid (AA) can re-reduce blue TMBox and yellow overoxidized TMBox to colorless TMB, while it cannot re-reduce diazotized TMBox to colorless TMB (the concentrations of nitrite and AA are 100 μM). Figure S14: Nitrite can trigger the oxidation and diazotization of TMB (the concentration of nitrite is 100 μM). Figure S15: Response comparison of various species toward the hollow MnFeO+TMB system (the concentrations of various species are 50 μM). Figure S16: Change of the ratiometric colorimetric signal over the reaction time of TMBox and nitrite. Figure S17: (A) displays UV-vis spectra of the TMB+NO_2_^−^ system with nitrite at various levels (the concentration of nitrite increases from 0 μM to 233.3 μM), and (B) shows the linear relationship between absorbance (439 nm) and nitrite concentration. Figure S18: The linear relationship between absorbance (540 nm) and nitrite concentration obtained by the commercial kit, Table S1: Comparison of kinetic parameters of oxidase-mimicking hollow MnFeO catalyzing the TMB oxidation reaction with that of other oxidase mimics. Table S2: Comparison of our ratiometric colorimetric assay with previously reported methods using TMB as a reagent for nitrite detection. Table S3: Comparison of our ratiometric colorimetric assay with previously reported nitrite detection methods.
