# Supplementary material for: Ratiometric Colorimetric Detection of Nitrite Realized by Stringing Nanozyme Catalysis and Diazotization Together
Source: Biosensors (Basel). 2021 Aug 18;11(8):280. doi: 10.3390/bios11080280 (PMC8394333; doi:10.3390/bios11080280)
Supplement: Supplementary file 1 [file biosensors-11-00280-s001.zip › biosensors-1327207-supplementary.pdf]

biosensors

MDPI

Supplementary Information

Ratiometric Colorimetric Detection of Nitrite Realized by Stringing Nanozyme Catalysis and Diazotization Together

Mengzhu Wang [1], Peng Liu [1], Hengjia Zhu [2], Bangxiang Liu [1] and Xiangheng Niu [1,2,3,4,*]

[1] Institute of Green Chemistry and Chemical Technology, School of Chemistry and Chemical Engineering, Jiangsu University, Zhenjiang 212013, China; wangmengzhu2018@126.com (M.W.); lp18852858622@163.com (P.L.); lbx15912338469@163.com (B.L.)
[2] School of Agricultural Engineering, Jiangsu University, Zhenjiang 212013, China; 18260622650@163.com
[3] Key Laboratory of Functional Molecular Solids, Ministry of Education, Anhui Normal University, Wuhu 241002, China
[4] State Key Laboratory of Urban Water Resource and Environment, Harbin Institute of Technology, Harbin 150090, China
* Correspondence: niuxiangheng@ujs.edu.cn

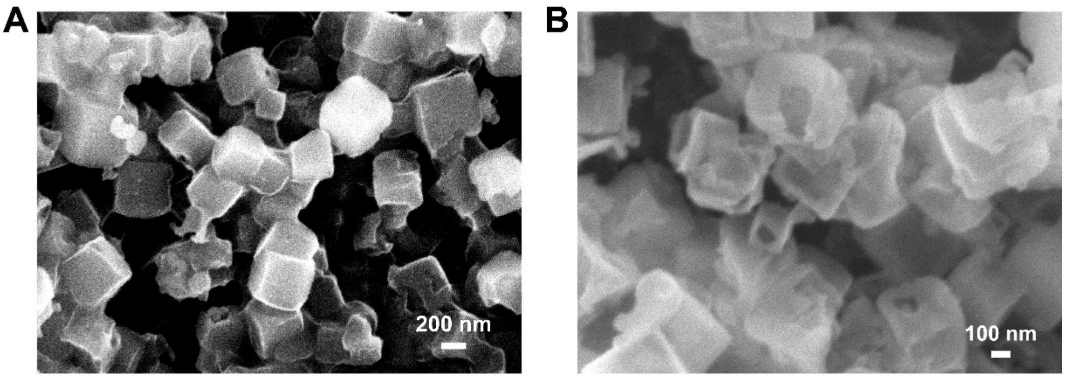

Figure S1. SEM images of Mn-Fe PBA (**A**) and hollow Mn-Fe PBA (**B**).

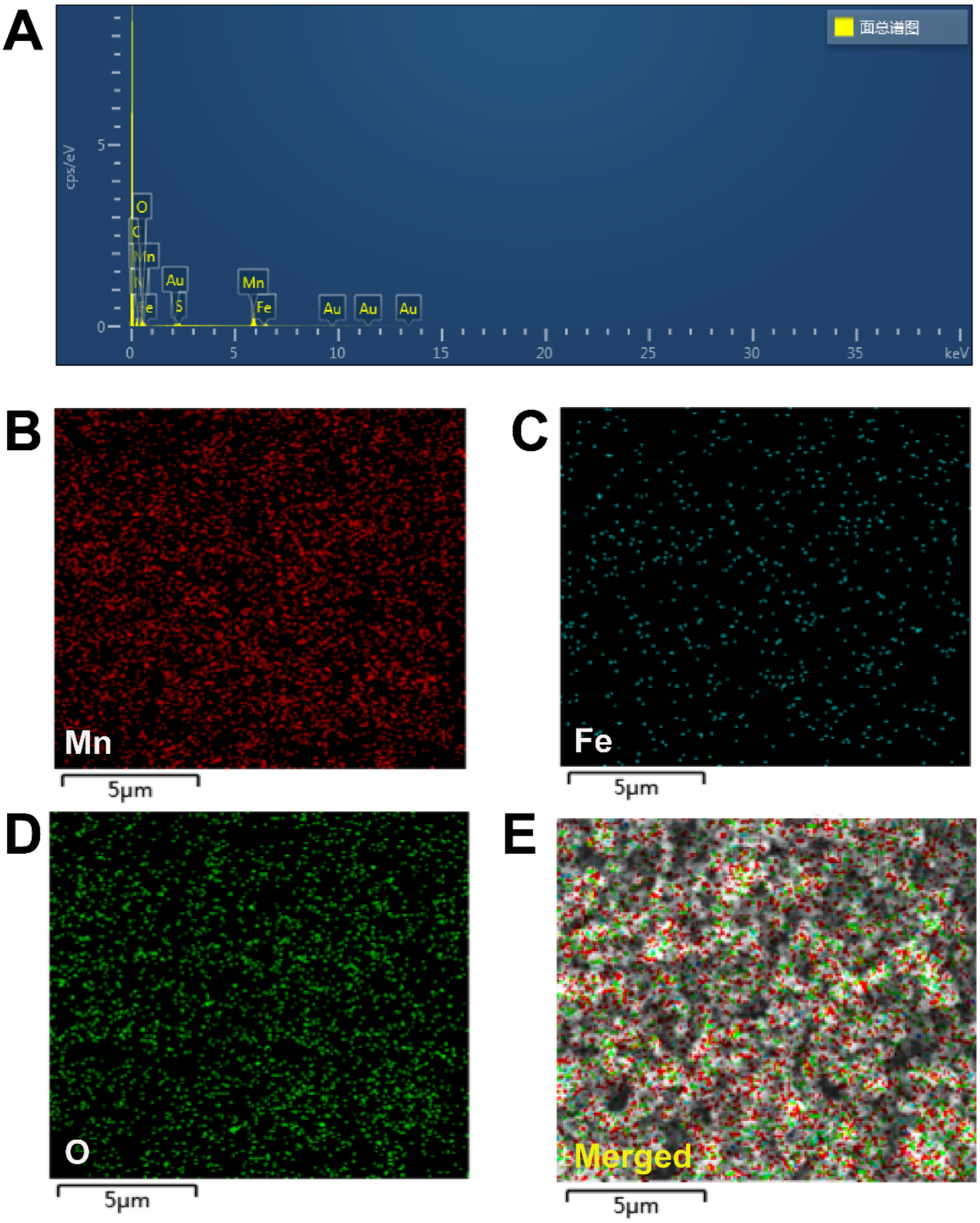

Figure S2. EDS (**A**) and elemental mapping images (**B–E**) of hollow MnFeO.

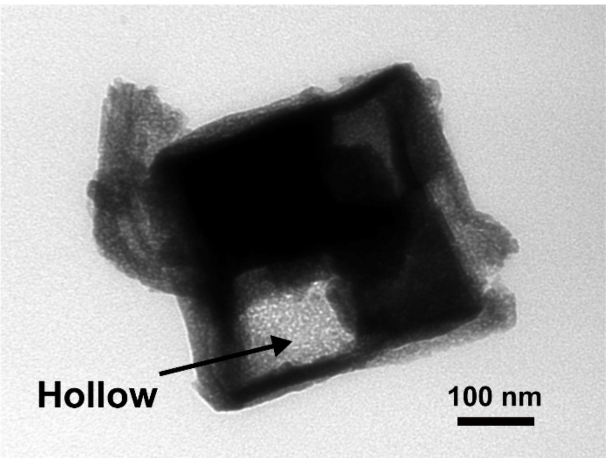

Figure S3. TEM image of hollow MnFeO.

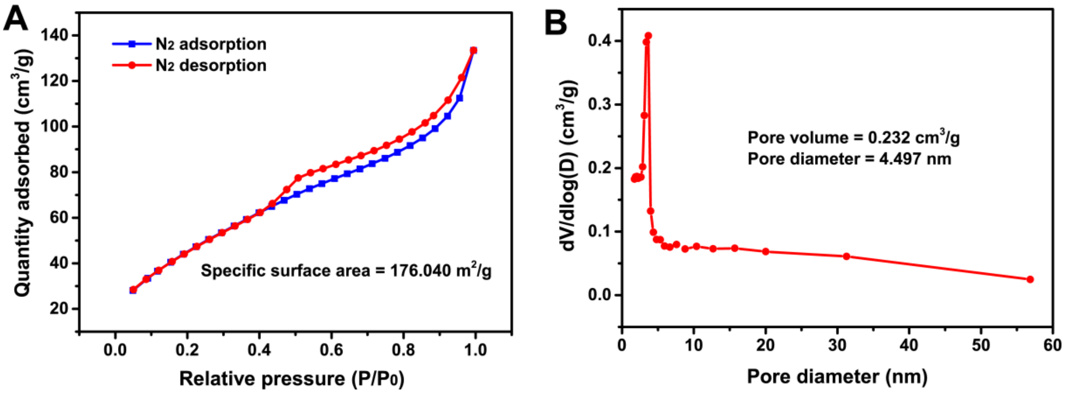

Figure S4. N_2 adsorption/desorption curves (**A**) of hollow MnFeO and its pore size distribution (**B**).

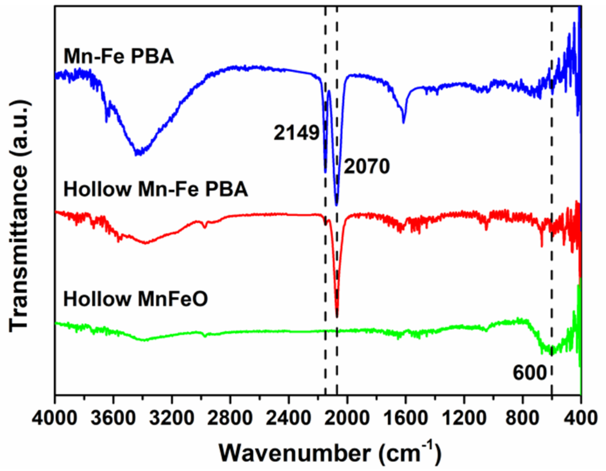

Figure S5. Comparison of FTIR spectra of different materials.

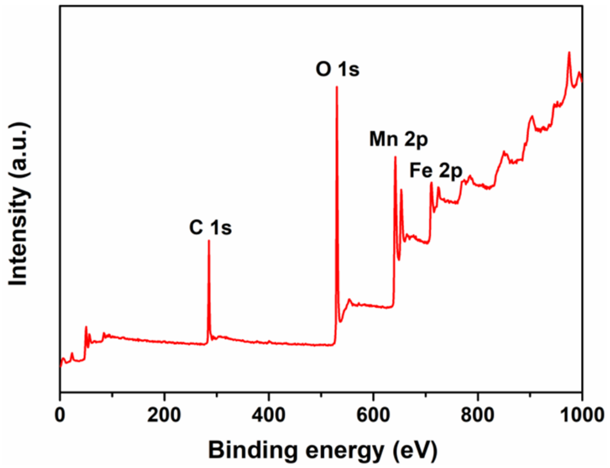

Figure S6. Full XPS of hollow MnFeO.

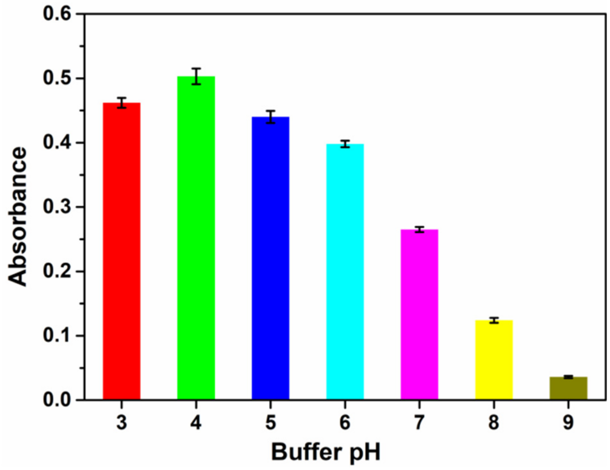

Figure S7. Effect of buffer pH on hollow MnFeO catalyzing the TMB chromogenic reaction.

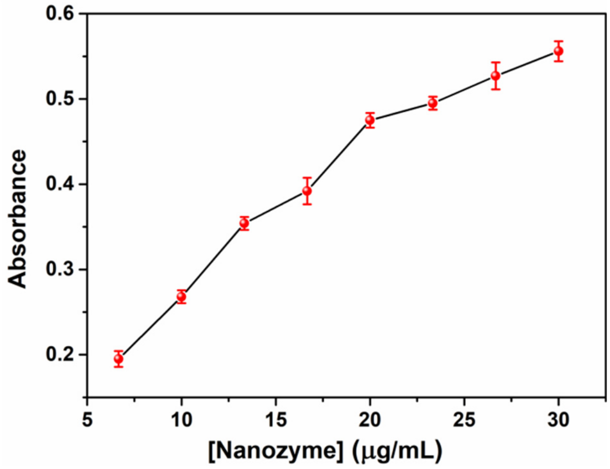

Figure S8. The absorbance (652 nm) of the hollow MnFeO+TMB system increases along with the amount of hollow MnFeO used.

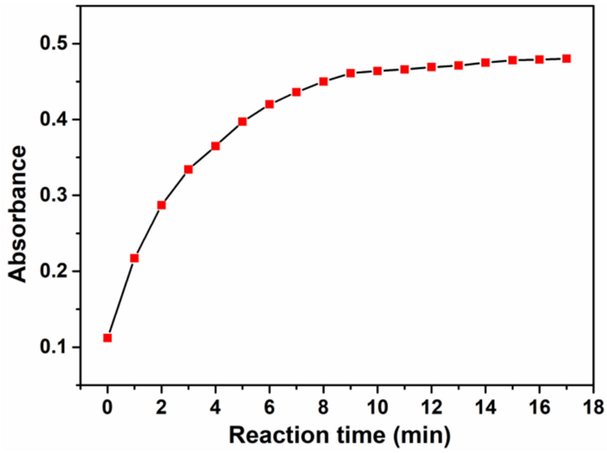

Figure S9. Absorbance (652 nm) change of the hollow MnFeO+TMB system over reaction time.

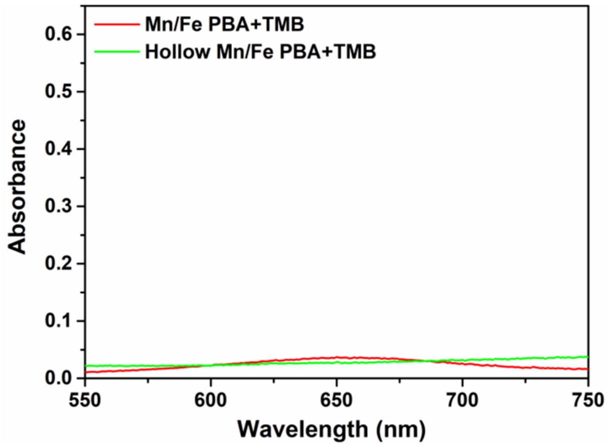

Figure S10. Neither Mn-Fe PBA nor hollow Mn-Fe PBA shows oxidase-like activity to catalyze the TMB chromogenic reaction.

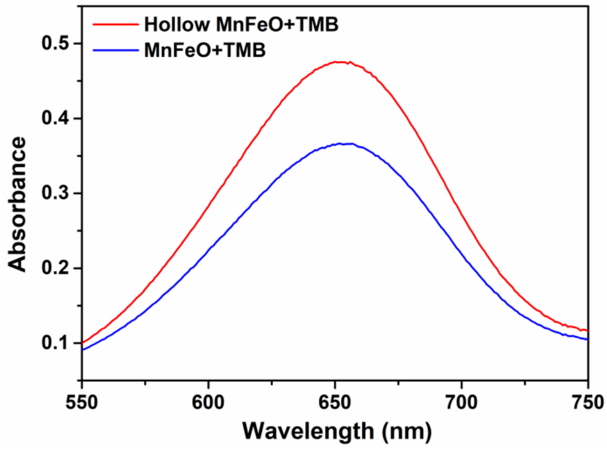

Figure S11. Oxidase-like activity comparison of MnFeO and hollow MnFeO catalyzing the TMB chromogenic reaction.

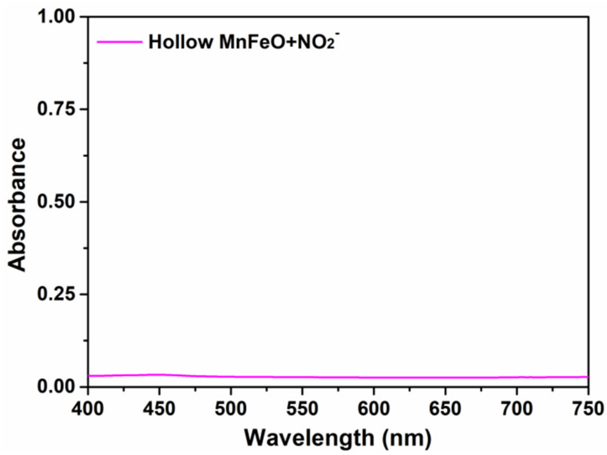

Figure S12. No chromogenic reaction occurs between hollow MnFeO and nitrite.

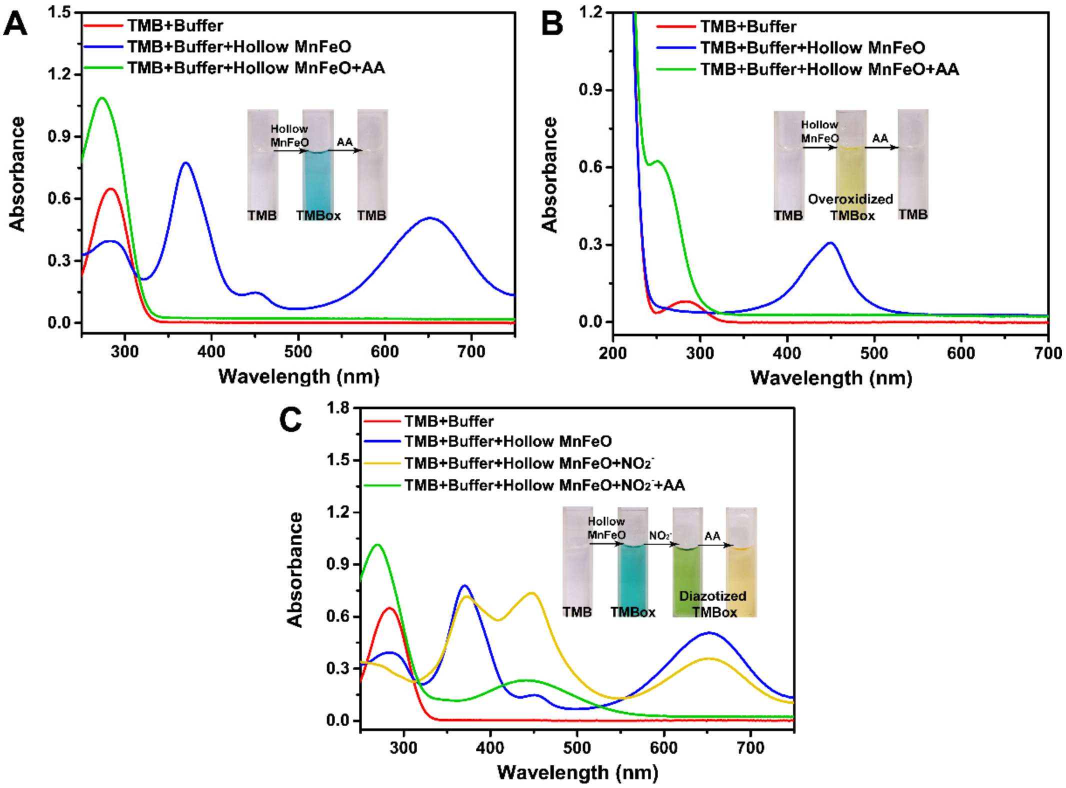

Figure S13. Ascorbic acid (AA) can re-reduce blue TMBox and yellow overoxidized TMBox to colorless TMB, while it cannot re-reduce diazotized TMBox to colorless TMB (the concentrations of nitrite and AA are 100 μM).

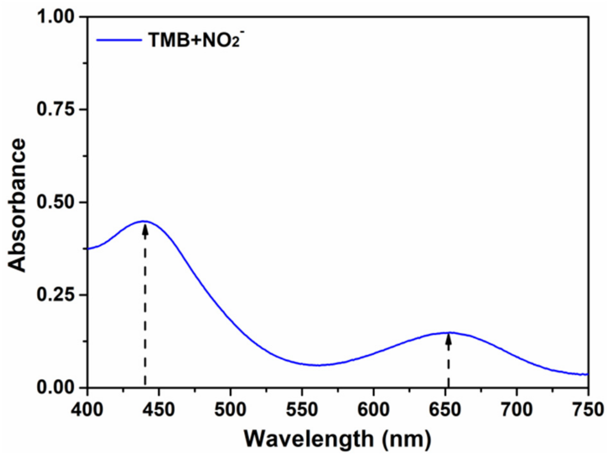

Figure S14. Nitrite can trigger the oxidation and diazotization of TMB (the concentration of nitrite is 100 μM).

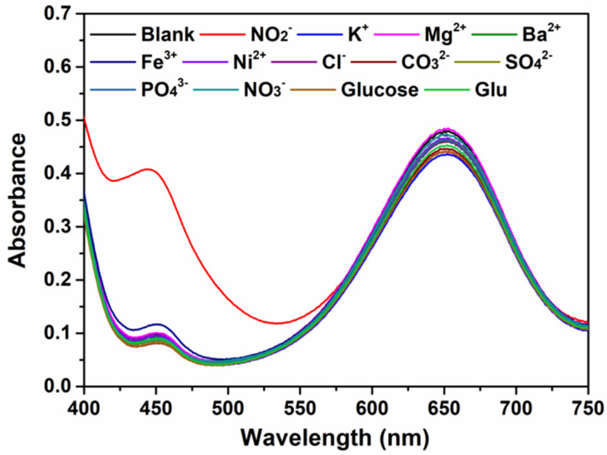

Figure S15. Response comparison of various species toward the hollow MnFeO+TMB system (the concentrations of various species are 50 μM).

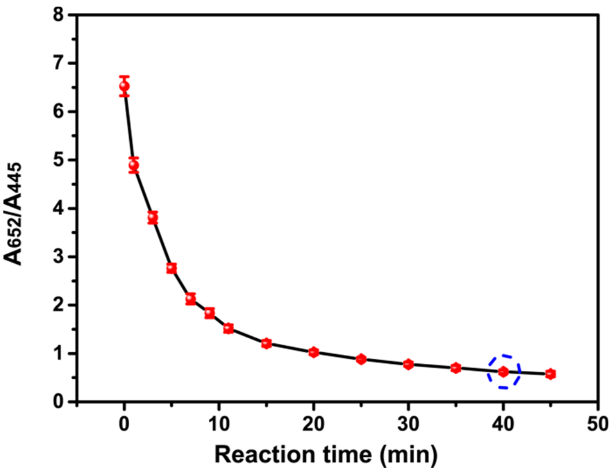

Figure S16. Change of the ratiometric colorimetric signal over the reaction time of TMBox and nitrite.

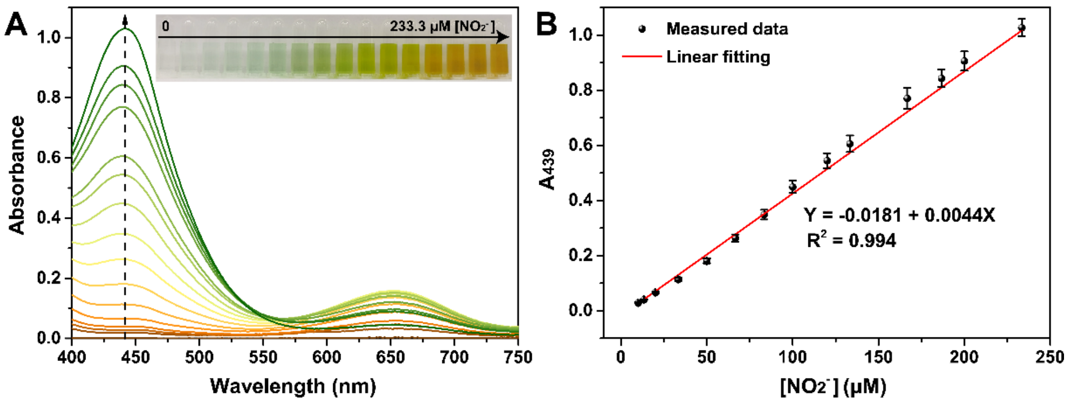

Figure S17. (**A**) displays UV-vis spectra of the TMB+NO_2^- system with nitrite at various levels (the concentration of nitrite increases from 0 μM to 233.3 μM), and (**B**) shows the linear relationship between absorbance (439 nm) and nitrite concentration.

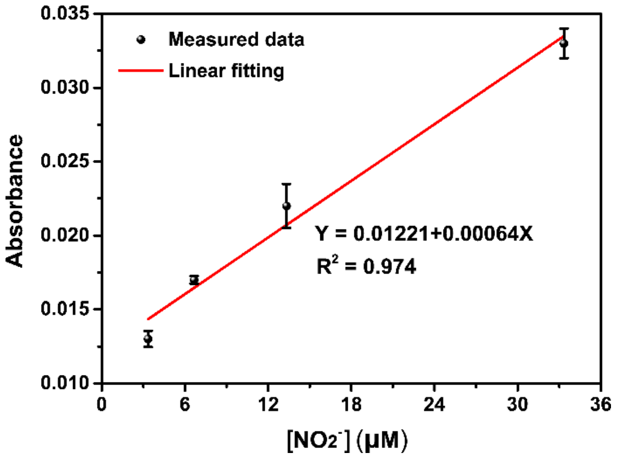

Figure S18. The linear relationship between absorbance (540 nm) and nitrite concentration obtained by the commercial kit.

Table S1. Comparison of kinetic parameters of oxidase-mimicking hollow MnFeO catalyzing the TMB oxidation reaction with that of other oxidase mimics.

Material	Substrate	K_m (mM)	V_{max} (×10^{-7}, M/s)	Reference
Hollow MnFeO	TMB	0.27	4.23	Our work
CeO_2	TMB	0.8-3.8	0.03-0.07	[1]
Pt nanoclusters	TMB	0.63	27	[2]
OV-Mn_3O_4 NFs	TMB	1.66	0.397	[3]
Ag_3PO_4	TMB	0.255	0.826	[4]
Fe–N/C	TMB	0.94	5.98	[5]

Table S2. Comparison of our ratiometric colorimetric assay with previously reported methods using TMB as a reagent for nitrite detection.

Principle	Detection Mode	Detection Range (μM)	LOD (μM)	Ref.
Oxidase-like catalysis	Colorimetric<br>Electrochemical	10-500<br>2.5-5700	2<br>0.7	[6]
Nitrite reductase-like catalysis	Colorimetric	100-5000	4.6	[7]
Diazo-coupling	Colorimetric	1-75	0.73	[8]
Oxidation	Colorimetric	10-440	2.34	[9]
Diazotization	Colorimetric	10-233.3	0.9	
Cascade of oxidase-like catalysis and diazotization	Ratiometric colorimetric	3.3-133.3	0.2	Our work

Table S3. Comparison of our ratiometric colorimetric assay with previously reported nitrite detection methods.

Principle	Detection Mode	Detection Range (μM)	LOD (μM)	Detection Time (min)	Ref.
Reduction	Colorimetric	0-30	0.1	90	[10]
Self-coupling diazotization	Colorimetric	2-40	0.12	100	[11]
Diazo-coupling	Colorimetric	0.3-22	0.149	35	[12]
Diazo-coupling	Colorimetric	22-30	22	25	[13]
Reduction	Fluorescent	0-60	0.07	5	[14]
Diazotization	Fluorescent	0.05-10	0.012	30	[15]
Diazo-coupling	Thread-based analytical device	50-1000	25	50	[16]
Cascade of oxidase-like catalysis and diazotization	Ratiometric colorimetric	3.3-133.3	0.2	55	Our work

References

1. A. Asati, S. Santra, C. Kaittanis, S. Nath, J.M. Perez, Oxidase-like activity of polymer-coated cerium oxide nanoparticles, Angew. Chem. Int. Ed. 48 (2009) 2308-2312.
2. C.J. Yu, T.H. Chen, J.Y. Jiang, W.L. Tseng, Lysozyme-directed synthesis of platinum nanoclusters as a mimic oxidase, Nanoscale 6 (2014) 9618-9624.
3. W.H. Lu, J. Chen, L.S. Kong, F. Zhu, Z.Y. Feng, J.H. Zhan, Oxygen vacancies modulation Mn_3O_4 nanozyme with enhanced oxidase-mimicking performance for L-cysteine detection, Sens. Actuators B Chem. 333 (2021) 129560.
4. P. Liu, X. Li, X.C. Xu, X.H. Niu, M.Z. Wang, H.J. Zhu, J.M. Pan, Analyte-triggered oxidase-mimetic activity loss of Ag_3PO_4/UiO-66 enables colorimetric detection of malathion completely free from bioenzymes, Sens. Actuators B Chem. 338 (2021) 129866.
5. Q.M. Chen, S.Q. Li, Y. Liu, X.D. Zhang, Y. Tang, H.X. Chai, Y.M. Huang, Size-controllable Fe-N/C single-atom nanozyme with exceptional oxidase-like activity for sensitive detection of alkaline phosphatase, Sens. Actuators B Chem. 305 (2020) 127511.
6. L. Liu, J. Du, W.E. Liu, Y.L. Guo, G.F. Wu, W.N. Qi, X.Q. Lu, Enhanced His@AuNCs oxidase-like activity by reduced graphene oxide and its application for colorimetric and electrochemical detection of nitrite, Anal. Bioanal. Chem. 411 (2019) 2189-2200.
7. O. Adegoke, S. Zolotovskaya, A. Abdolvand, N.N. Daeid, Rapid and highly selective colorimetric detection of nitrite based on the catalytic-enhanced reaction of mimetic Au nanoparticle-CeO_2 nanoparticle-graphene oxide hybrid nanozyme, Talanta 224 (2021) 121875.
8. Y.Y. Chen, C.X. Zhao, G.Z. Yue, Z.P. Yang, Y.Y. Wang, H.B. Rao, W. Zhang, B. Jin, X.X. Wang, A highly selective chromogenic probe for the detection of nitrite in food samples, Food Chem. 317 (2020) 126361.
9. H.H. Wang, X. Jing, X.Y. Bi, B. Bai, X.W. Wang, Quantitative detection of nitrite in food samples based on digital image colourimetry by smartphone, ChemistrySelect 5 (2020) 9952-9956.
10. J.Y. Du, M.X. Zhao, W. Huang, Y.Q. Deng, Y. He, Visual colorimetric detection of tin(II) and nitrite using a molybdenum oxide nanomaterial-based three-input logic gate, Anal. Bioanal. Chem. 410 (2018) 4519-4526.
11. H.H. Wang, N.W. Wan, L. Ma, Z.Q. Wang, B.D. Cui, W.Y. Han, Y.Z. Chen, A novel and simple spectrophotometric method for detection of nitrite in water, Analyst 143 (2018) 4555-4558.

12. M.H. Ibrahim, Z.H. Xue, H.I. Abdu, M.I. Shinger, A.M. Idris, M.M. Edris, D.L. Shan, X.Q. Lu, Sensitive and selective colorimetric nitrite ion assay using silver nanoparticles easily synthesized and stabilized by AHNDMS and functionalized with PABA, Nanoscale Adv. 1 (2019) 1207-1214.
13. W.L. Daniel, M.S. Han, J.S. Lee, C.A. Mirkin, Colorimetric nitrite and nitrate detection with gold nanoparticle probes and kinetic end points, J. Am. Chem. Soc. 131 (2009) 6362-6363.
14. K. Chaiendoo, K. Ngamdee, W. Limbut, C. Saiyasombat, W. Busayaporn, S. Ittisanronnachai, V. Promarak, K. Promsuwan, P. Thavarungkul, P. Kanatharana, W. Ngeontae, Gold nanoparticle-based cascade reaction-triggered fluorogenicity for highly selective nitrite ion detection in forensic samples, Microchem. J. 168 (2021) 106470.
15. M.C. Rong, D.R. Wang, Y.Y. Li, Y.Z. Zhang, H.Y. Huang, R.F. Liu, X.Z. Deng, Green-emitting carbon dots as fluorescent probe for nitrite detection, J. Anal. Test. 5 (2021) 51-59.
16. P. Singhaphan, F. Unob. Thread-based platform for nitrite detection based on a modified Griess assay, Sens. Actuators B Chem. 327 (2021) 128938.
